# The complete plastome and phylogenetic analysis of *Zingiber ottensii* Valeton

**DOI:** 10.1080/23802359.2026.2622800

**Published:** 2026-02-10

**Authors:** Zhaofei Wang, Hualan Chen, Jinyong Ni, Gang Huang, Jia Dong, Honglei Li, Maoqin Xia

**Affiliations:** Chongqing University of Arts and Science, Yongchuan, Chongqing, China

**Keywords:** *Zingiber ottensii*, Zingiberaceae, plastome, phylogenetic analysis

## Abstract

*Zingiber ottensii* Valeton (1918) is a perennial herb in Zingiberaceae, which has important medicinal and edible values. Here, we first reported and characterized the complete chloroplast genome (plastome) of *Z. ottensii* using the whole-genome resequencing technology. The plastome was 169,569 bp in size and contained 138 genes, including 92 protein-coding genes, 38 tRNA genes, and 8 rRNA genes. It exhibits a typical quadripartite structure with a large single-copy region (LSC, 86,760 bp), a small single-copy region (SSC, 7,615 bp), and a pair of inverted repeats (IRs, 37,597 bp). Phylogenetic analysis confirmed that *Z. ottensii* belonged to *Zingiber* sect. *Zingiber* and revealed its close relationship to *Zingiber zerumbet* (L.) Roscoe ex Sm. These results provided essential genomic resources for future phylogenetic and evolutionary studies of *Zingiber* Mill.

## Introduction

*Zingiber ottensii* Valeton (1918) is a perennial herb in the family Zingiberaceae, distributed in Southeast Asia, including Indonesia (Java and Sumatra), Laos, Peninsular Malaysia, Myanmar, Thailand, and Vietnam (Aung and Tanaka 2019). This species is characterized by a dark purple rhizome and a broad, spindle-shaped inflorescence (Ly et al. 2016). It bears 2–3 flowers at a time; these flowers emerge from bracts and exhibit a color range from pale cream to light dark yellow. Given its long history of traditional medicinal use, *Z. ottensii* has become a focus of extensive research, primarily aiming to validate and exploit its potential therapeutic applications (Thitinarongwate et al. 2021; Panyajai et al. 2022). Specifically, studies have shown that *Z. ottensii* contains a range of bioactive compounds, including zerumbone, terpinene-4-ol, α-humulene, and sabinene, which are directly responsible for the plant’s reported medicinal properties (Chen et al. 2025). Among these metabolites, zerumbone has been identified as the primary active compound, recognized for its potential analgesic and anti-inflammatory effects (Wongpia et al. 2025). However, despite its significant medicinal potential, the genetic resources of *Z. ottensii* remain limited, posing challenges for further exploration and sustainable utilization of this valuable species.

Chloroplast genome (plastome) is typically characterized by a conserved structural organization and a size variation between 120 and 160 kb. It consists of a single circular chromosome featuring a quadripartite architecture, wherein two inverted repeat (IR) regions flank and separate a large single-copy (LSC) region and a small single-copy (SSC) region (Daniell et al. 2016; De Vries and Archibald 2018). Intercompartmental gene transfer among the plastome, mitochondrial genome, and nuclear genome has been documented in plants (e.g. Fang et al. 2024; Bai et al. 2025). These findings imply that structural variations in the plastome are associated with speciation and can serve as a valuable source of evolutionary insights (Cosner et al. 2004). With rapid development of the second-generation sequencing technology, the plastome information was widely used for studying taxonomy, phylogeny and evolution in plants (Zhang et al. 2022; Du et al. 2025; Su et al. 2025; Yu and He 2025).

Although several studies have focused on the evolution of plastome in plants of *Zingiber* (e.g. Jiang et al. 2023; Tian et al. 2023; Xia et al. 2024), the number of reported plastomes to date accounts for less than 1/4 of the total species (*c.* 31/141) in this genus (Xia et al. 2024). Recent phylogenomic studies utilizing comprehensive plastome datasets have yielded significant insights into the phylogenetic relationships within the Zingiberaceae family. Notably, these findings have facilitated a more precise delineation of the sections within the genus *Zingiber* (Lu et al. 2025). The plastome of *Z. ottensii* and its evolutionary position in *Zingiber* remains unresolved. In this study, we sequenced and annotated the plastome sequence of *Z. ottensii* using the whole-genome resequencing technology. Comparative plastome analysis and phylogenetic analysis were conducted to reveal its chloroplast structural feature and phylogenetic position.

## Materials and methods

### Plant sampling, DNA extraction and sequencing

**Figure 1.**
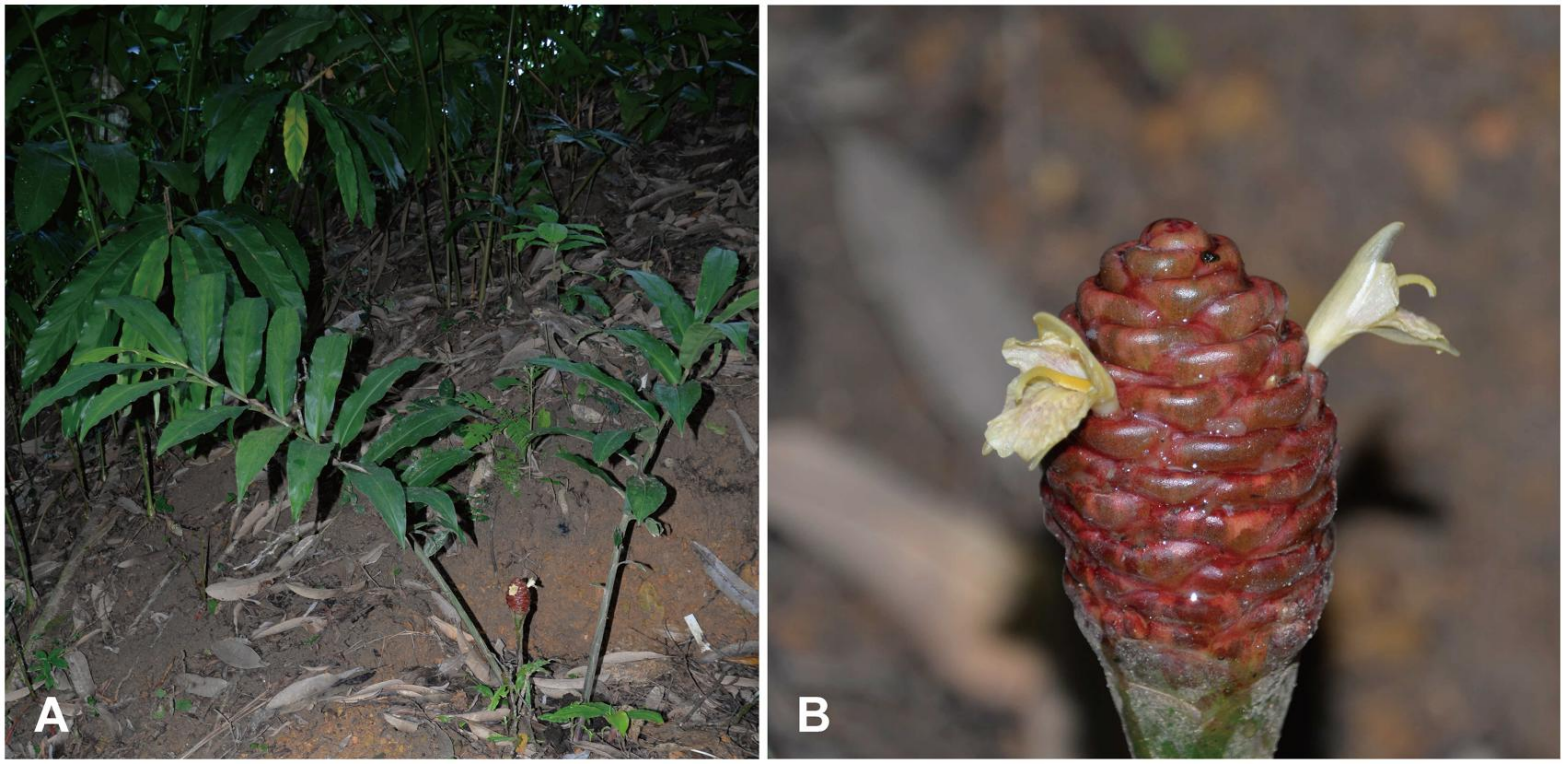
The feature of *Zingiber ottensii* used in this study. (A) The cultivated *Z. ottensii* in Xishuangbanna Tropical Botanical Garden of Chinese Academy of Sciences. A rhizomatous, perennial herb with leafy shoots reaching 1.5 m in height. The rhizome is purplish inside and has a strong pungent odor. (B) Inflorescence of *Z. ottensii*. Spiciform, borne on separate scapes (25–40 cm). Bracts are obovate, 4 cm, bright red with incurved tips. Bracteoles are linear, 3 cm. The spike is ellipsoidal to cylindrical, 10–12 cm. Image credit: Hualan Chen (13189457624@163.com). Photographed by Hualan Chen.

The samples of *Z. ottensii* (Figure 1) were collected from Xishuangbanna Tropical Botanical Garden of Chinese Academy of Sciences (101.2700°E, 21.9204°N). Voucher specimen (yunnan031-1) of this species was deposited at Plant Herbarium of Chongqing University of Arts and Sciences (contact person and email: Maoqin Xia, xiamq@cqwu.edu.cn). Genomic DNA (gDNA) was extracted from approximately 50 mg of leaf tissue using the Plant Genomic DNA Kit (TIANGEN, Beijing, China). Following quality control, gDNA was fragmented using ultrasonication with the Covaris E220 system (Covaris, Brighton, UK). Fragment sizes (300 to 500 bp) were end-repaired, A-tailed, and ligated with indexed adaptors at both ends. The resulting products were amplified by PCR and circularized to generate a single-stranded circular (ssCir) library. This library was then subjected to rolling circle amplification (RCA) to produce DNA nanoballs (DNBs), which were subsequently sequenced on the MGI-DNBSEQ platform (Shenzhen, China) to obtain 150 bp paired-end reads.

### Assembly and annotation of the plastome

The chloroplastome of *Z. ottensii* was assembled with GetOrganelle v1.7.7 (Jin et al. 2020). The k-mers (-k) were set as 21,45,65,85,105. Then, we annotated the assembled sequence using CPGAVAS2 (Shi et al. 2019) with default parameters and with the plastome of *Zingiber zerumbet* (L.) Roscoe ex Sm. (GenBank accession number: NC_049006.1) as the reference. Additionally, the promoter and terminator of each protein-coding gene sequence (CDS) were manually checked in Geneious v2023.2.1 (Biomatters Ltd.). Finally, the plastome of *Z. ottensii* was visualized using CPGView (Liu et al. 2023).

### Phylogenetic analysis

The phylogenetic tree was constructed based on 76 protein-coding genes (PCGs) shared among 17 plastomes of the Zingiberaceae species. *Costus tonkinensis* Gagnep. and *Hellenia speciosa* (J. Koenig) S. R. Dutta (Costaceae) were used as outgroups. The sequences alignments were generated using MAFFT v7.526 (Katoh and Standley 2013). Ambiguously aligned and low-mutation sites were filtered using Gblocks v0.91b with default settings (Talavera and Castresana 2007). The nucleotide substitution model was estimated using ModelFinder (Kalyaanamoorthy et al. 2017), and the best-fit model (TVM+F + I + G4) was selected based on the Bayesian Information Criterion (BIC). A maximum-likelihood (ML) tree was constructed using IQ-Tree v2.3.6 (Nguyen et al. 2015) with 5000 bootstrap replicates, and visualized in iTOL v6 (https://itol.embl.de/) (Letunic and Bork 2021).

## Result

**Figure 2.**
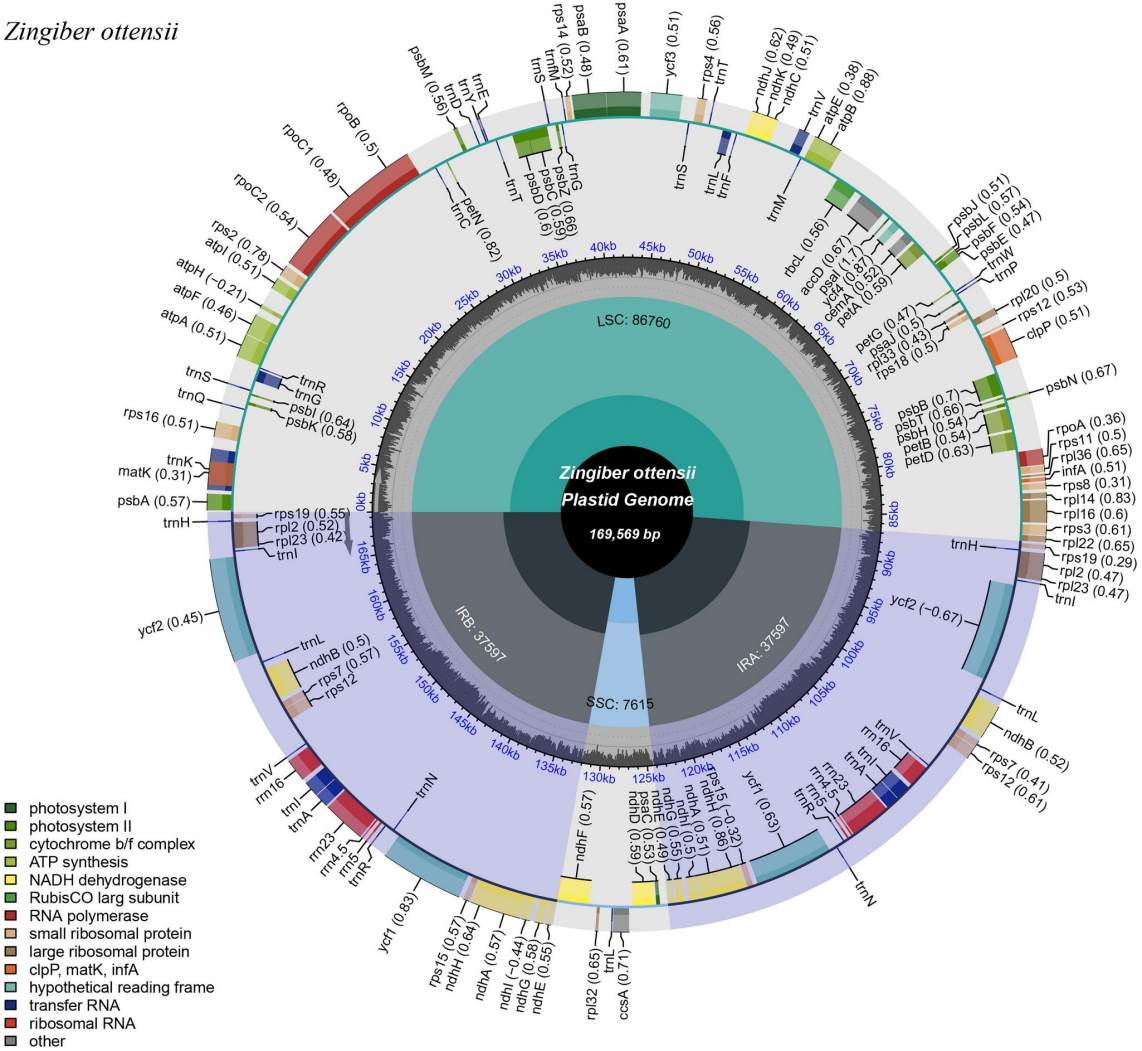
A circular map of the plastome of *Z. ottensii*. The transcription directions of the genes in the inner and outer regions are clockwise and anticlockwise, respectively. Each functional gene group is color-coded for clarity. The inner circle represents GC content with darker gray shades, while lighter gray shades indicate the AT content.

The plastome of *Z. ottensii* is 169,569 bp in length and exhibits a typical quadripartite structure, comprising a LSC region of 86,760 bp, a SSC region of 7,615 bp, and a pair of IRs of 37,597 bp (Figure 2). The coverage depth ranged from 56✕ to 338✕, with a mean depth of 234.2✕, confirming the reliability of the assembled genome (Figure S1). The overall GC content of the genome is 35.8%, while the IRs show a significantly higher GC content (38.4%) compared to the LSC (34.1%) and SSC (30.2%) regions. After manual examination of the annotations, a total of 138 genes were identified, including 92 protein-coding genes (CDS), 38 tRNA genes, and 8 rRNA genes. Among these, *ndh*E, *ndh*G, *ndh*H, *ndh*I, *rpl*23, *rps*12, *rps*15, *rps*19, *rps*7, *ycf*1, *ycf*2, *rpl*2, *ndh*A, *ndh*B, *trn*S-GGA, *trn*N-GUU, *trn*R-ACG, *trn*A-UGC, *trn*I-GAU, *trn*V-GAC, *trn*L-CAA, *trn*I-CAU, *trn*H-GUG, *rrn*5S, *rrn*4.5S, *rrn*23S, and *rrn*16S are multi-copy genes, each with two copies. Twelve cis-splicing genes were identified, including nine genes (*rps*16, *atp*F, *rpo*C1, *pet*B, *pet*D, *rpl*16, *rpl*2, *ndh*B, *ndh*A) with a single intron, and three (*ycf*3, *acc*D, *clp*P) with two introns (Figure S2). Additionally, the *rps*12 gene is trans-spliced, with its exons located at separate positions within the genome, which are joined through a complex splicing process to generate the mature mRNA (Figure S3).

**Figure 3.**
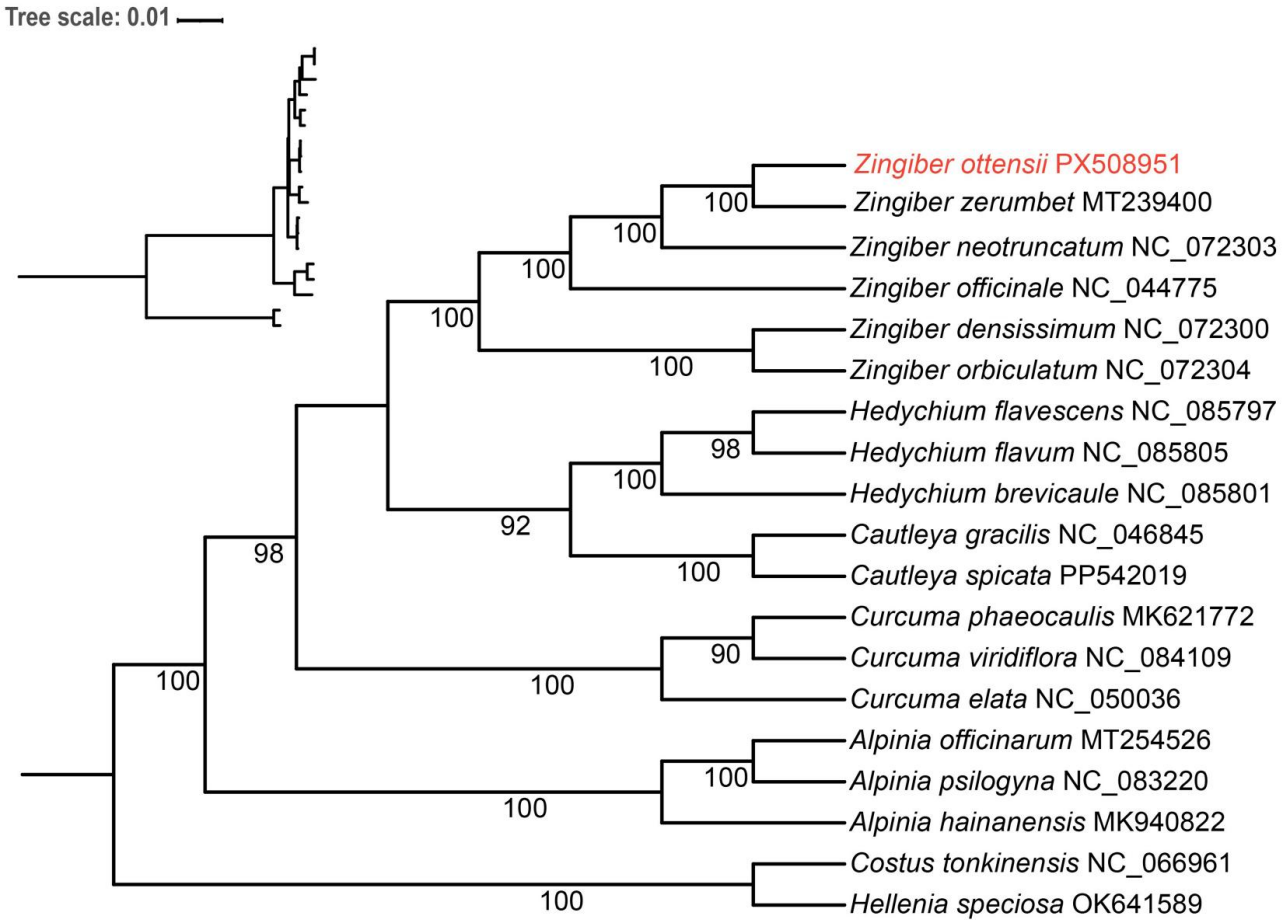
Maximum likelihood (ML) phylogenetic tree constructed using 76 protein-coding genes from the plastome of *Z. ottensii* and 19 plastomes from the Zingiberaceae and Costaceae families. The accession numbers of used sequences follow the species names, and the newly sequenced genome is shown in red font. Sequences used for tree construction were as follows: *Zingiber zerumbet* MT239400 (Qi et al. 2020); *Zingiber neotruncatum* NC_072303 (Jiang et al. 2023); *Zingiber officinale* NC_044775 (Cui et al. 2019); *Zingiber densissimum* NC_072300 (Jiang et al. 2023); *Zingiber orbiculatum* NC_072304 (Jiang et al. 2023); *Hedychium flavescens* NC_085797 (Li et al. 2023); *Hedychium flavum* NC_085805 (Li et al. 2023); *Hedychium brevicaule* NC_085801 (Li et al. 2023); *Cautleya gracilis* NC_046845 (Li and Wang 2019); *Cautleya spicata* PP542019 (unpublished); *Curcuma phaeocaulis* MK621772 (Gui et al. 2020); *Curcuma viridiflora* NC_084109 (Lin et al. 2024); *Curcuma elata* NC_050036 (unpublished); *Alpinia officinarum* MT254526 (Huang 2021); *Alpinia psilogyna* NC_083220 (unpublished); *Alpinia hainanensis* MK940822 (unpublished); *Costus tonkinensis* NC_066961 (unpublished); *Hellenia speciosa* OK641589 (unpublished).

Under the optimal model (TVM+F + I + G4) selected by BIC, the ML phylogenetic tree received high bootstrap support (BS) for most nodes (Figure 3). Phylogenetic analysis indicated that all *Zingiber* species formed a monophyletic clade (BS = 100) and established a sister relationship with *Hedychium* and *Cautleya* (BS = 92). Within the *Zingiber*, *Z. densissimum* and *Z. orbiculatum* were sister to each other with high supported values (BS = 100), and then grouped with the clade of *Z. officinale*, *Z. neotruncatum*, *Z. zerumbet* and *Z. ottensii. Zingiber ottensii* showed a sister relationship to *Z. zerumbet* (BS = 100).

## Discussion and conclusions

This study first reported the complete plastome of *Z. ottensii*. With a total length of 169,569 bp, this genome was notably the largest one among all previously reported plastomes within *Zingiber* (Qi et al. 2020; Jiang et al. 2023; Wang et al. 2024; Xia et al. 2024). We proposed that the relatively long length of its plastome is attributed to the expansion of the IR regions, a phenomenon that has also been reported in its closely related species *Z. zerumbet* (GenBank accession number: MT239400; Qi et al. 2020). The inflorescences of *Z. ottensii* and *Z. zerumbet* are characterized by a spike on a long erect peduncle, which is a distinctive feature of species classified under *Zingiber* sect. *Zingiber* (Bentham, 1873). In contrast, species belonging to *Zingiber* sect. *Cryptanthium* typically exhibit inflorescences with short or sprawling peduncles. Based on morphological characteristics, *Z. ottensii*, *Z. zerumbet*, *Z. neotruncatum*, and *Z. officinale* are classified under *Z*. sect. *Zingiber*, while *Z. densissimum* and *Z. orbiculatum* fall under *Z*. sect. *Cryptanthium*. The phylogenetic tree constructed in this study also supports this classification, confirming that *Z. ottensii* is the number of the *Z*. sect. *Zingiber* and, in the current sampling, forms a sister relationship with *Z. zerumbet*. These findings not only validate the morphological classification but also demonstrate the monophyly of the two sections within *Zingiber*.

To further elucidate the phylogenetic position of *Z. ottensii*, we referenced a recent comprehensive phylogenomic study of the Zingiberaceae family (Lu et al. 2025). Although *Z. ottensii* was not included in that analysis, Lu et al. (2025) robustly supported the monophyly of a core *Zingiber* clade (excluding *Z. ellipticum*), a finding that aligns closely with our results. Additionally, their study confirmed the sister relationship between *Z*. sect. *Cryptanthium* and *Z*. sect. *Zingiber*, consistent with the topology observed in our analysis, wherein *Z. densissimum* and *Z. orbiculatum* (*Z*. sect. *Cryptanthium*) serve as a sister group to species within *Z*. sect. *Zingiber*. Within this context, the strong placement of *Z. ottensii* as sister to *Z. zerumbet* in *Z*. sect. *Zingiber* provides vital species-level resolution. By contributing the first plastome sequence for *Z. ottensii*, our study enhances genomic representation within this section and further corroborates the infrageneric phylogenetic framework established by broader analyses.

In conclusion, the plastome sequence of *Z. ottensii* not only enriched genomic resources for *Zingiber*, but also offers valuable data for future phylogenetic studies this genus.

## Supplementary Material

Supplemental Material 1.docx

Figure.zip

Supplementary File 2.xlsx

## Data Availability

The genome sequence data that support the findings of this study is available in GenBank of NCBI (https://www.ncbi.nlm.nih.gov/) under accession number PX508951. The associated BioProject, BioSample, and SRA numbers are PRJNA1344276, SAMN52633237, and SRR35768944, respectively.
