# Supplementary material for: The complete plastome and phylogenetic analysis of Zingiber ottensii Valeton
Source: Mitochondrial DNA B Resour. 2026 Feb 10;11(3):398–403. doi: 10.1080/23802359.2026.2622800 (PMC12895869; doi:10.1080/23802359.2026.2622800)
Supplement: Supplemental Material 1.docx [file TMDN_A_2622800_SM7999.docx]

**Supplemental material**

**
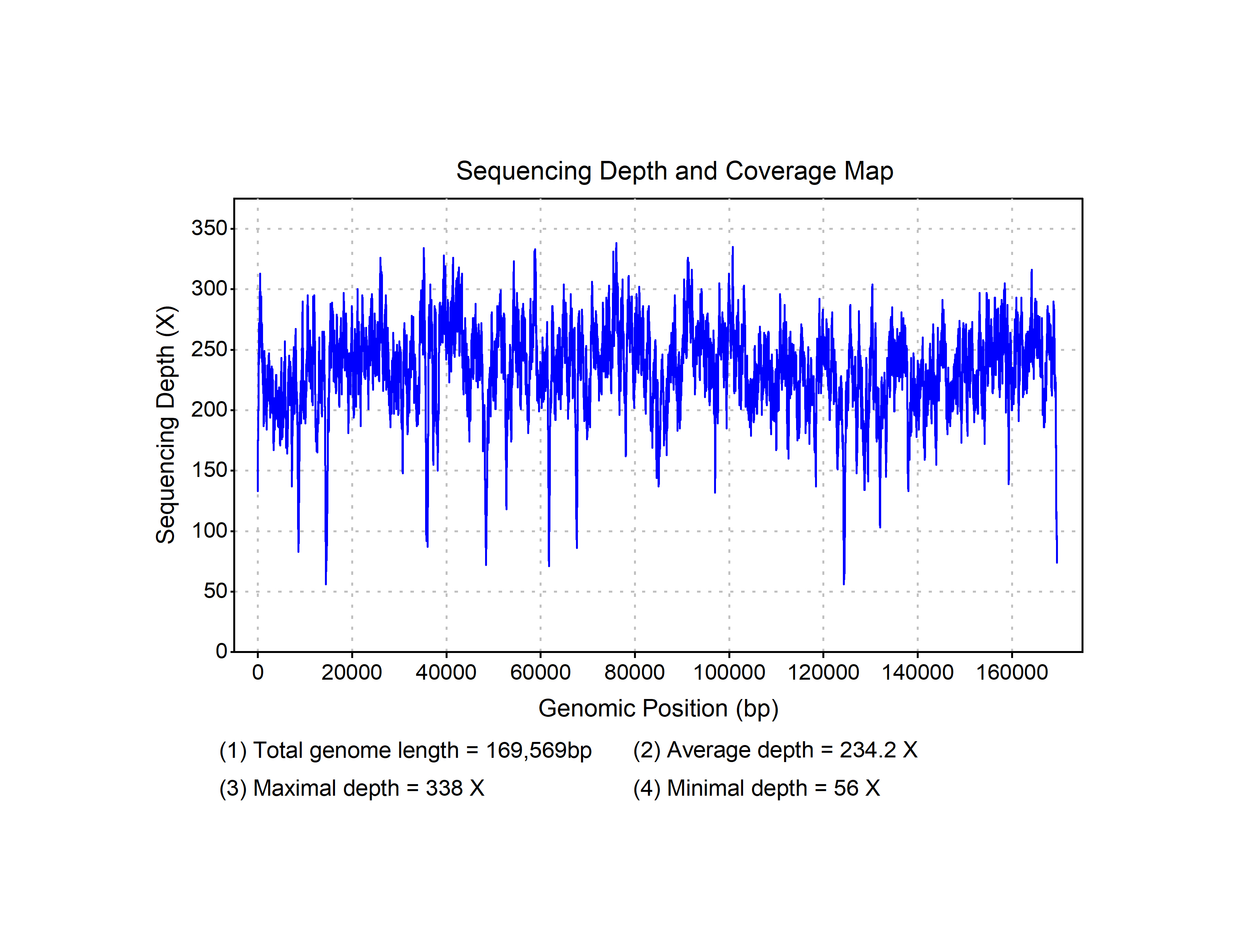
**

**Figure S1.** Sequencing depth and coverage map of the *Zingiber ottensii* chloroplast genome assembly. The horizontal axis represents the base positions along the plastome, while the vertical axis indicates the sequencing depth at each base position.


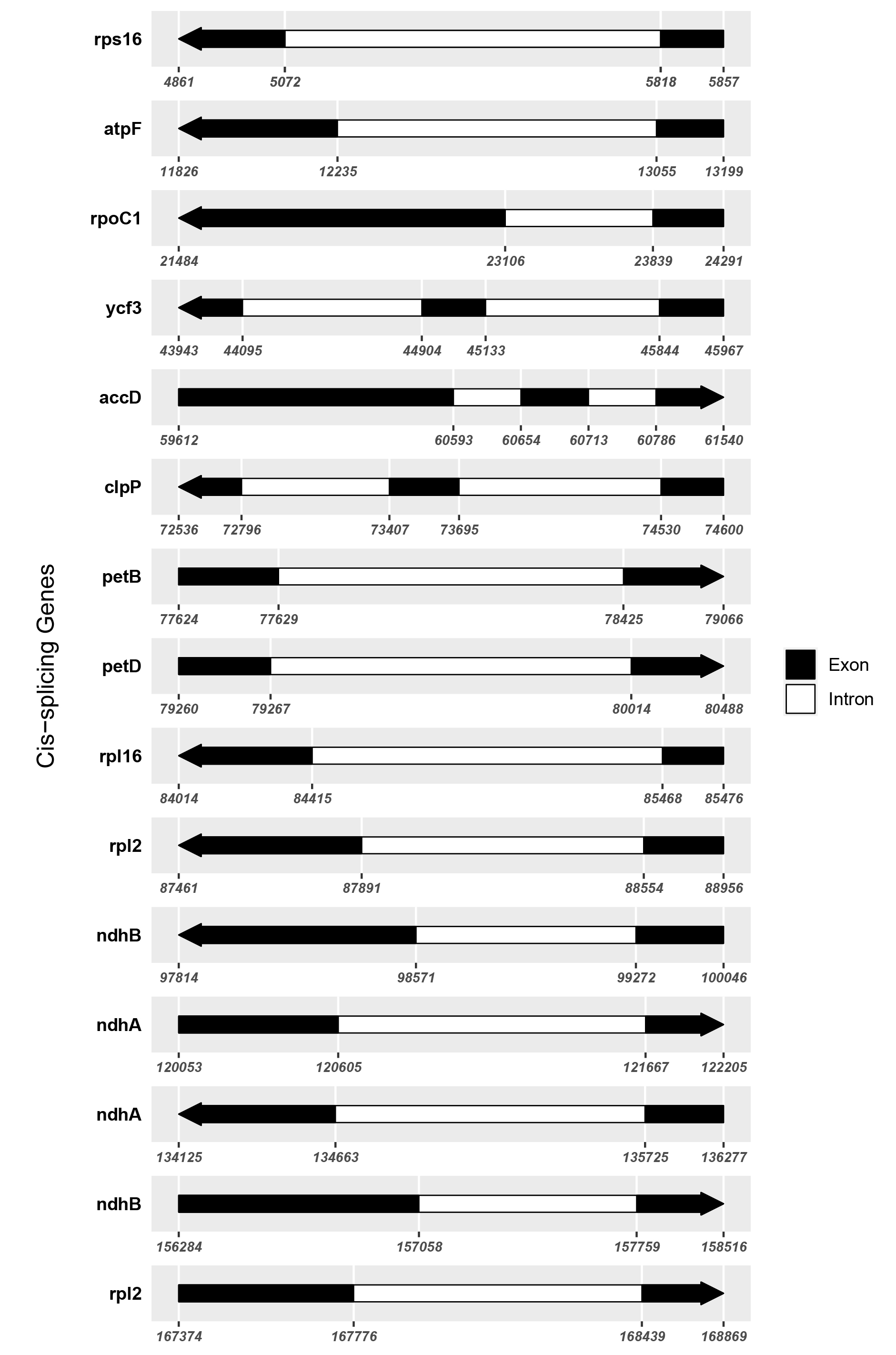


**Figure S2.** Schematic representation of the cis-splicing genes in the chloroplast genome of *Zingiber ottensii*. Exons are shown in black, introns in white, and arrows indicate the gene’s sense direction. This figure was enerated using CPGView (Liu et al. 2023).


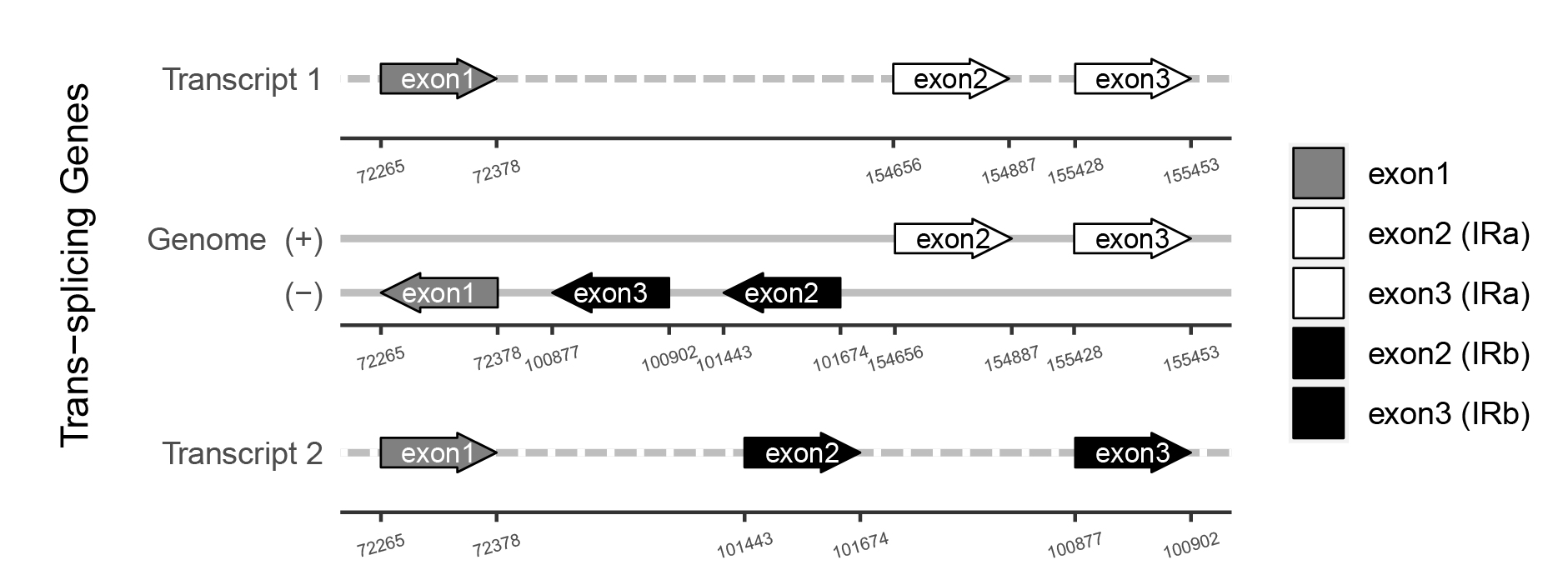


**Figure S3.** Schematic representation of the trans-splicing gene *rps12* in the chloroplast genome of *Zingiber ottensii*. The arrow indicates the gene's sense direction. This figure was enerated using CPGView (Liu et al. 2023).
