# Supplementary figures and images for: The complete plastome and phylogenetic analysis of Zingiber ottensii Valeton
Source: Mitochondrial DNA B Resour. 2026 Feb 10;11(3):398–403. doi: 10.1080/23802359.2026.2622800 (PMC12895869; doi:10.1080/23802359.2026.2622800)

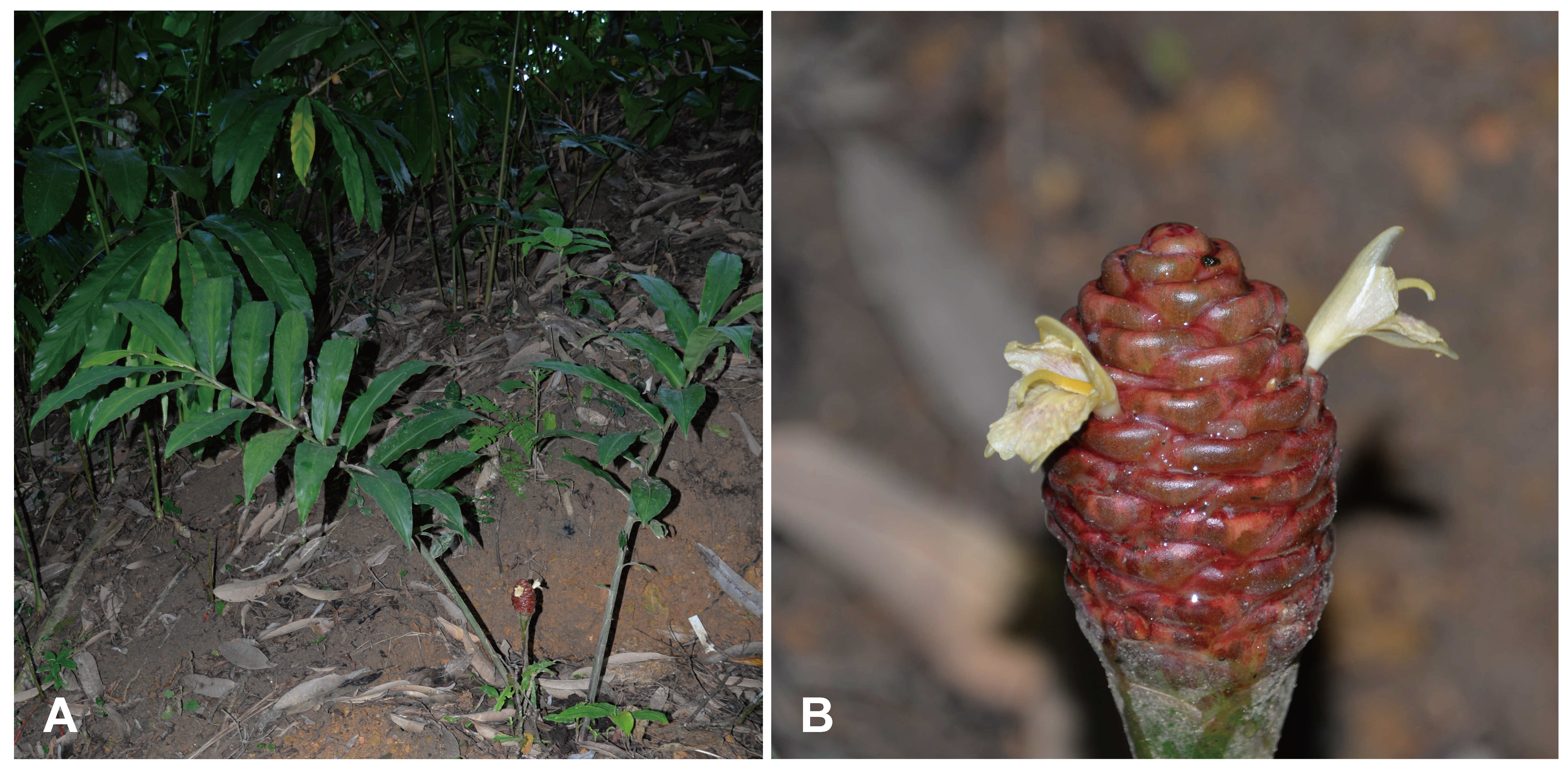

Supplement: Figure.zip [file TMDN_A_2622800_SM7998.zip › Figure/Figure 1.png]

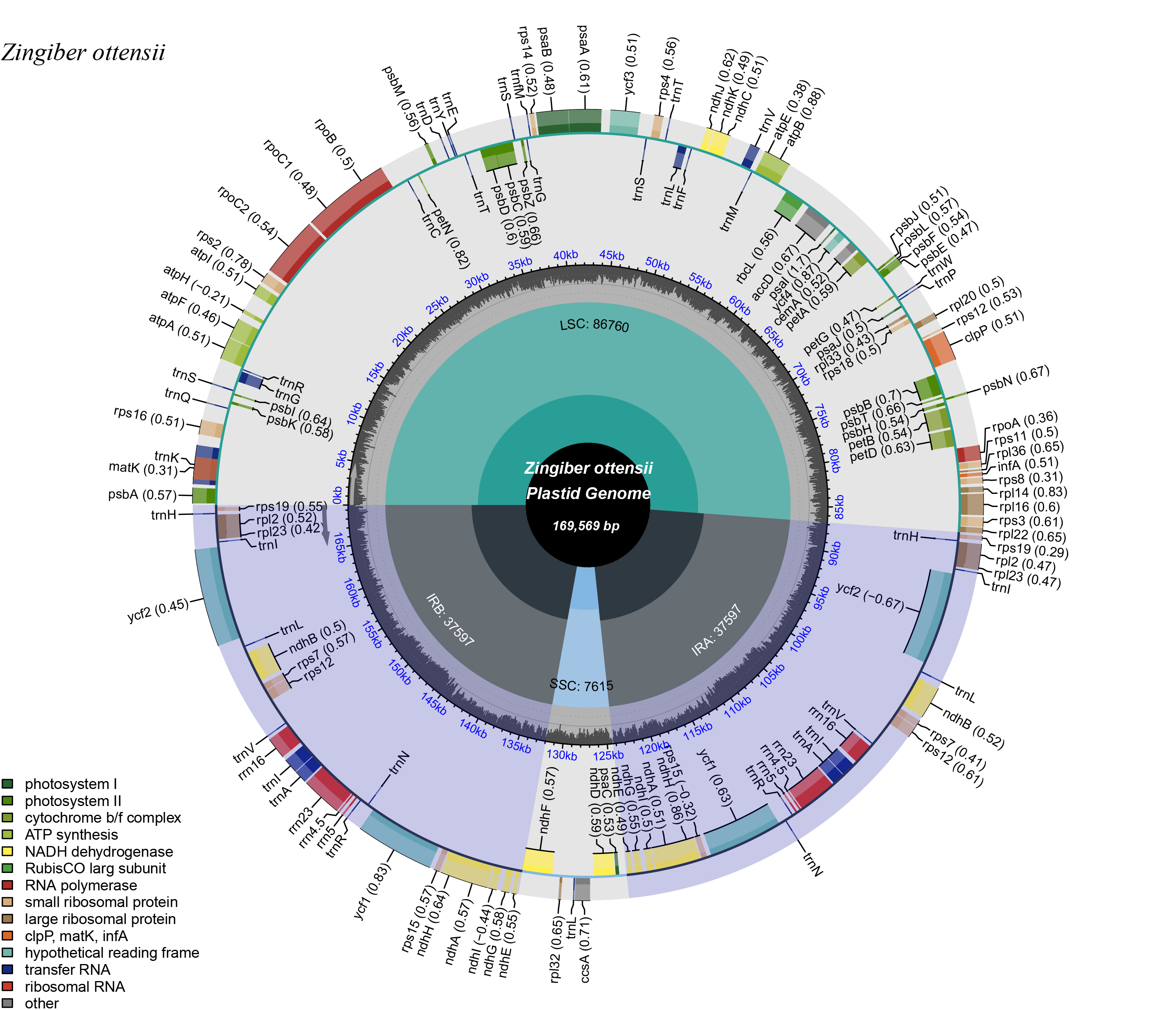

Supplement: Figure.zip [file TMDN_A_2622800_SM7998.zip › Figure/Figure 2.jpg]

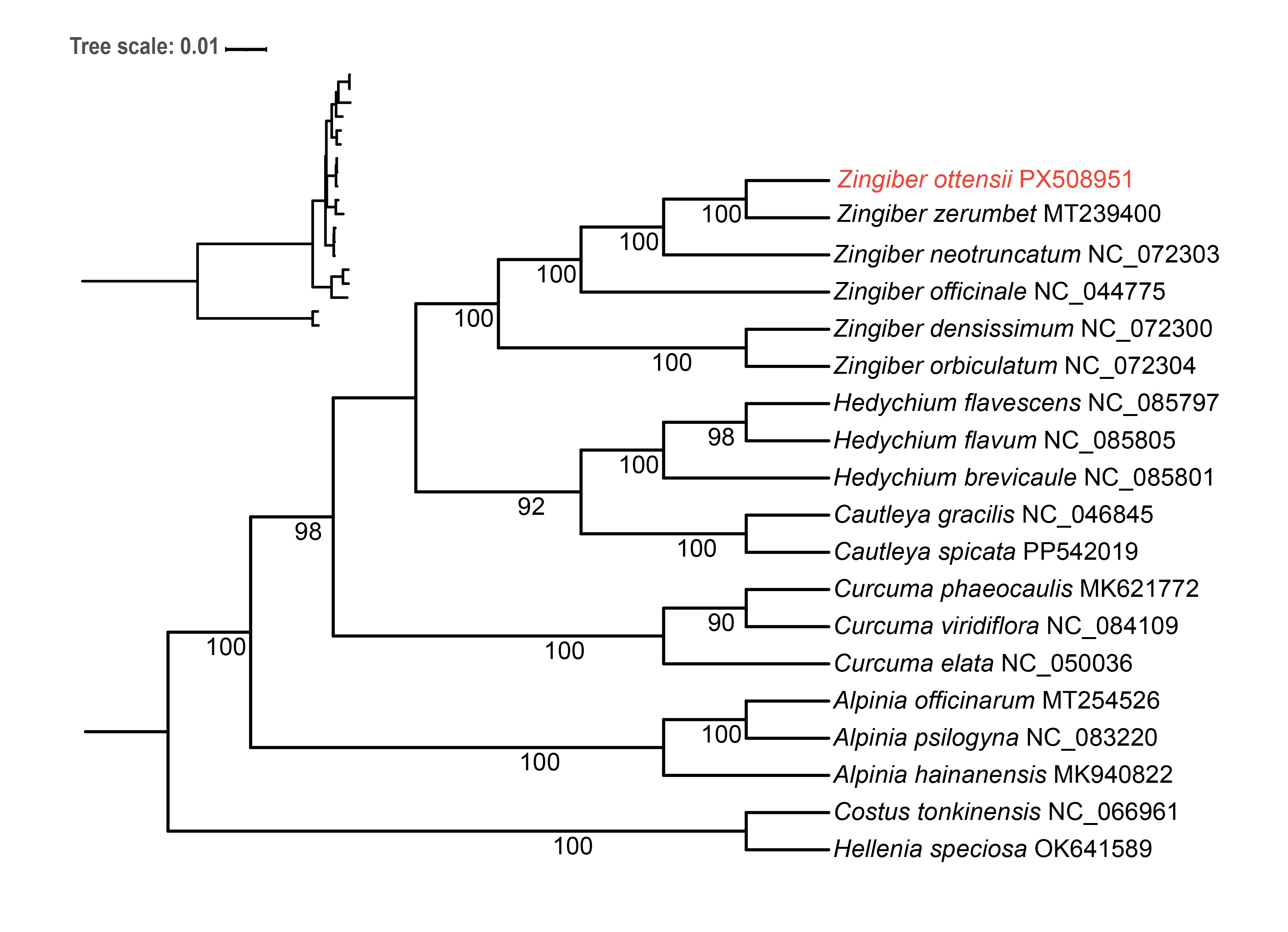

Supplement: Figure.zip [file TMDN_A_2622800_SM7998.zip › Figure/Figure 3.jpg]

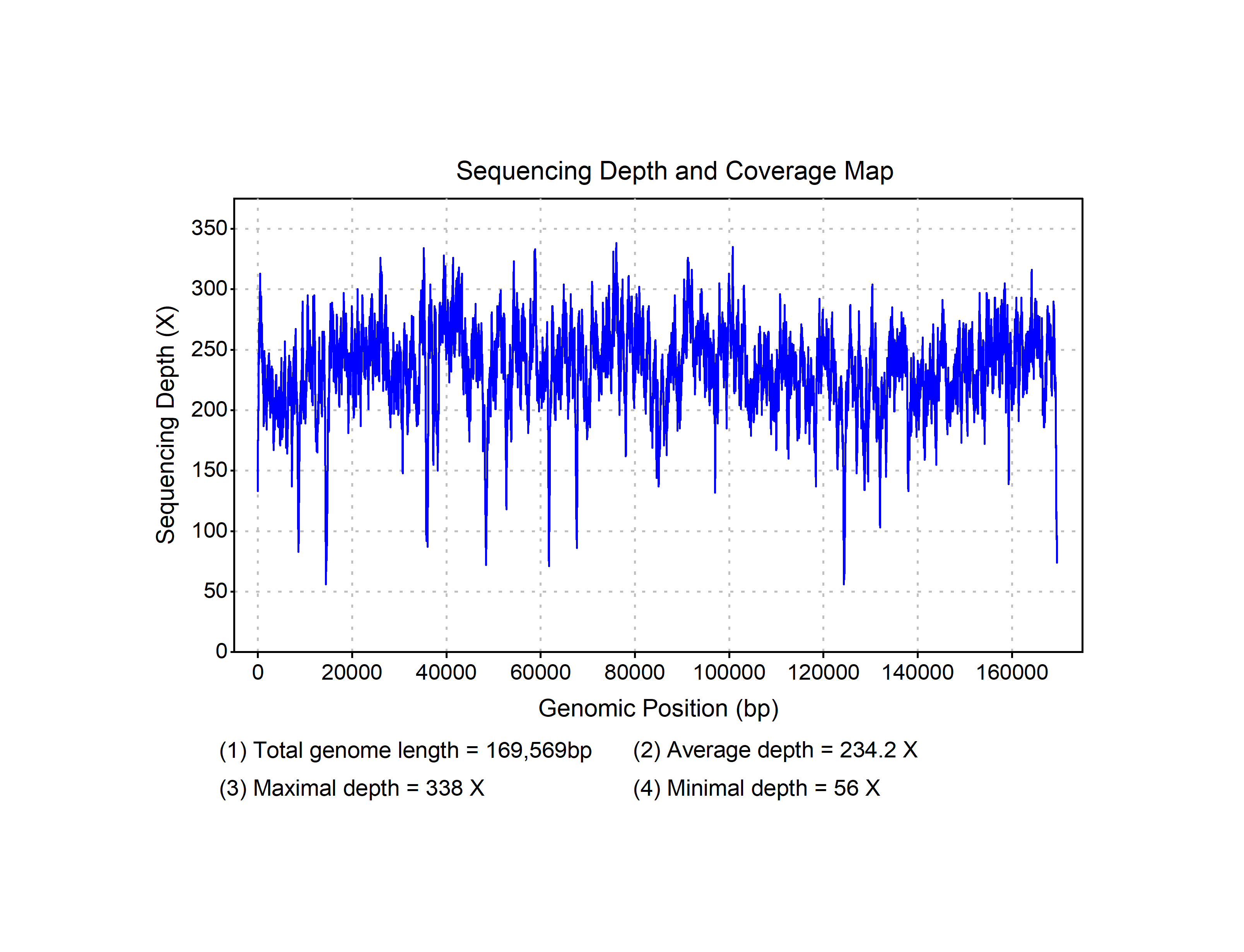

Supplement: Figure.zip [file TMDN_A_2622800_SM7998.zip › Figure/Figure S1.jpg]

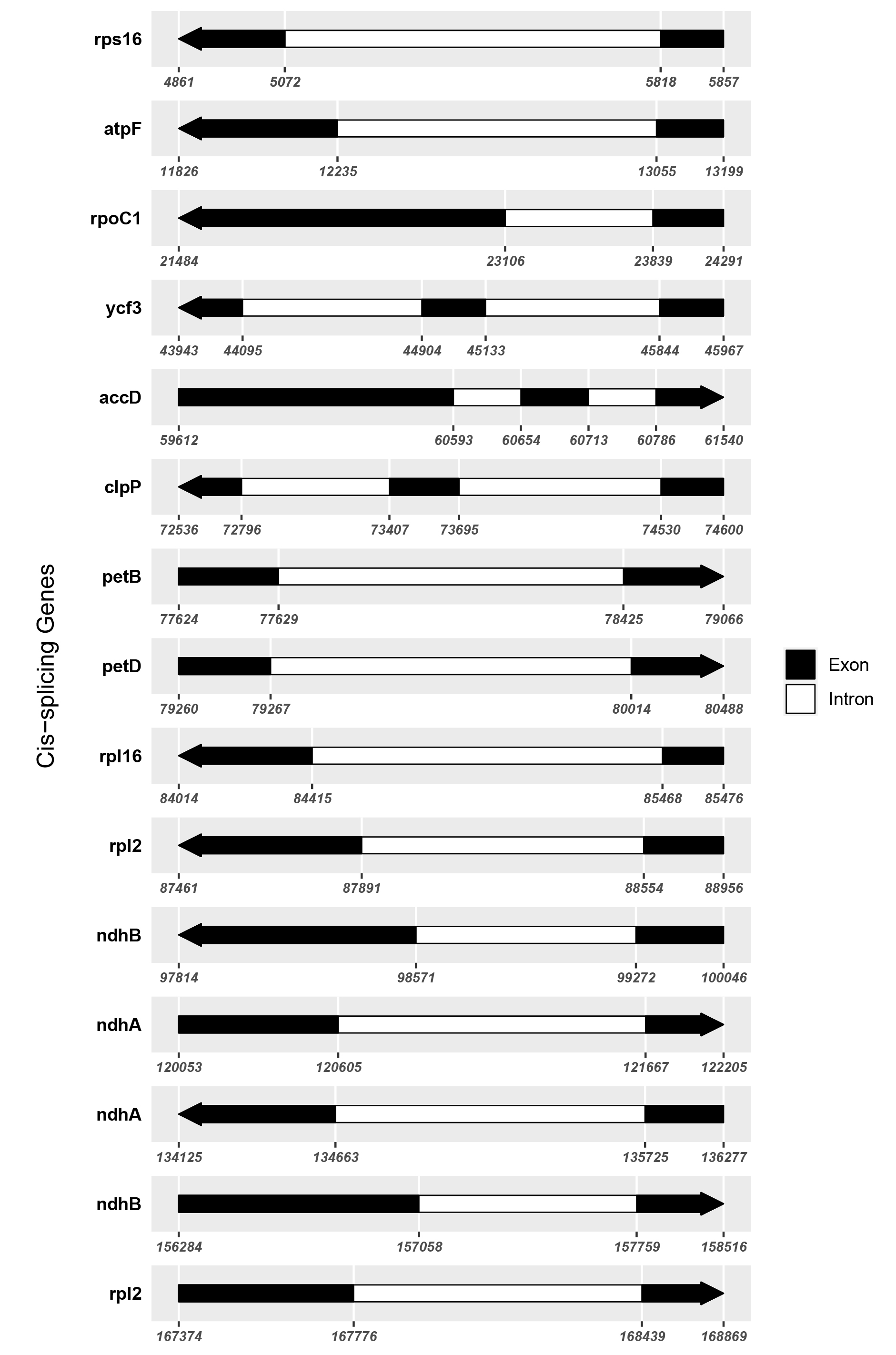

Supplement: Figure.zip [file TMDN_A_2622800_SM7998.zip › Figure/Figure S2.jpg]

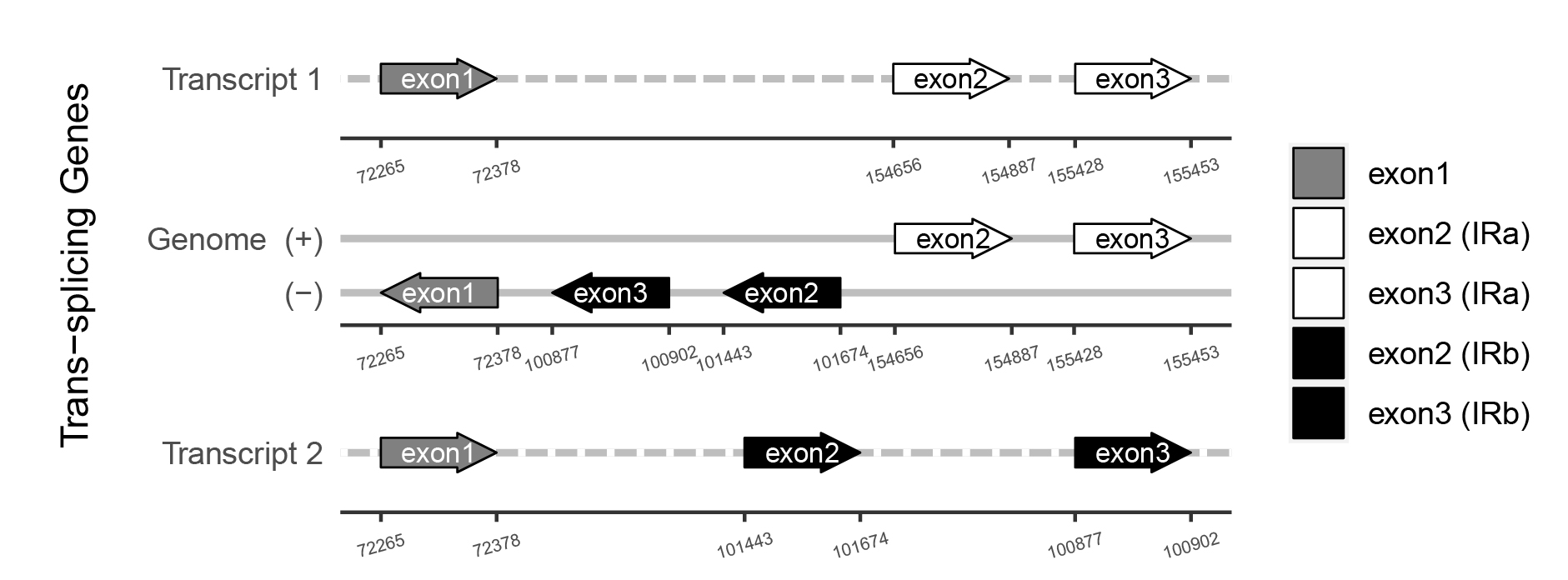

Supplement: Figure.zip [file TMDN_A_2622800_SM7998.zip › Figure/Figure S3.jpg]
